# Transforming tennis with artificial intelligence: a bibliometric review

**DOI:** 10.3389/fspor.2024.1456998

**Published:** 2024-12-23

**Authors:** Tatiana Sampaio, João P. Oliveira, Daniel A. Marinho, Henrique P. Neiva, Jorge E. Morais

**Affiliations:** ^1^ Department of Sports Sciences, University of Beira Interior, Covilhã, Portugal; ^2^ Research Centre in Sports, Health and Human Development (CIDESD), Covilhã, Portugal; ^3^ Research Centre for Active Living and WellBeing (LiveWell), Bragança, Portugal; ^4^ Department of Sports Sciences, Instituto Politécnico de Bragança, Bragança, Portugal

**Keywords:** machine learning, deep learning, artificial intelligence, sport, tennis

## Abstract

The aim of this study was to conduct a scoping and bibliometric review of articles using artificial intelligence (AI) in tennis. The analysis covered various aspects of tennis, including performance, health, match results, physiological data, tennis expenditure, and prize amounts. Articles on AI in tennis published until 2024 were retrieved from the Web of Science database. A total of 389 records were screened, and 108 articles were retained for analysis. The analysis identified intermittent gaps in publication output during certain intervals, notably in the years 2007–2008 and 2012–2013. From 2012 onward, there was a clear upward trend in publications and citations, peaking in 2022. The theme was led by China, the United States, and Australia. These countries maintain their status as the top contributors in terms of publications. The analysis of author collaborations revealed multiple clusters, with notable contributions from researchers in China, Australia, Japan, and the United States. This bibliometric review has elucidated the evolution of AI research in tennis, highlighting the countries and authors that have significantly contributed to this field over the years. The prediction model suggests that the number of articles and citations on this topic will continue to increase over the next decade (until 2034).

## 1 Introduction

Tennis has experienced significant changes in recent decades (1). This globally practiced sport enjoyed both recreationally and competitively, requires players to continually develop and master essential technical skills for effective performance during matches. As the world's second most popular sport, trailing only soccer, tennis is played in 195 countries and boasts an estimated 87 million fans — individuals who have played tennis at least once — representing about 1.17% of the global population (2, 3).

Historically, tennis was primarily a technical sport, with a strong emphasis on specific skills such as stroke techniques. However, it has evolved into a more dynamic and explosive sport, characterized by increased serve and stroke velocities, and significantly higher physical demands (4, 5). The physical fitness levels of tennis players are now critical in determining match outcomes, particularly in competitions where players have closely matched skill levels (6, 7). To compete at the highest level, athletes now require a holistic combination of speed, agility, and power, coupled with moderate to high aerobic capacity (1). Supporting these physical demands are critical cognitive and psychological processes (8, 9). Players must exhibit exceptional reactive abilities, anticipation skills, and decision-making skills while maintaining mental fortitude to cope with fatigue, the pressure of high-stakes points, and the draw of significant extrinsic rewards like ranking and lucrative endorsements (10–12). The stop-and-start nature of tennis competitions further adds to the complexity (5).

Computational intelligence has developed into a powerful instrument in recent years for optimizing athletic performance in a variety of sports. With huge possibilities for future growth in the upcoming years, interest in AI and its subcategories is expanding at an exponential rate (13).

Applied scientists find machine learning (ML) to be an effective instrument in this dynamic and data-rich environment (14, 15). In terms of both research and practical application, the USA has been recognized as a leader in the application of AI in sports. Artificial intelligence has been utilized for player and game analysis for several years, mostly in baseball and American football (16).

AI and its subcategories, ML and deep learning have both seen significant growth in recent years. Therefore, the term AI is used even when referring to ML or deep learning in individual publications (17). The vast majority agree that AI is just a machine-implemented form of human intelligence. An approach to AI called ML describes computer systems that can learn from experiences or examples without explicit programming. In the domain of automatic feature discovery through representation learning, deep learning has also become a key methodology for ML implementation as an artificial neural network-based method (17).

The availability (i.e., quality and quantity) of data, which has a significant impact on the performance of AI models, has been one of the causes supporting the exponential growth of AI in recent years, as has the ongoing enhancement of the related techniques (18). Additionally, many people are finding it easier and more accessible to create ML models thanks to user-friendly open-source programming libraries like PyTorch (19) and scikit-learn (20). These factors have made it possible for AI to gradually make progress into sports contexts and applications, opening up an array of new possibilities (21, 22).

A significant bibliographic dataset has resulted from the research situation about AI technologies in sports, which makes manual examination unfeasible. To identify and justify new research necessities, the relevant research and structures must be thoroughly examined to uncover existing knowledge, challenges, and research shortcomings.

Bibliometric analysis is a promising method that is growing to become increasingly significant in many areas of research. For instance, bibliometric analyses are used to investigate the intellectual structures of specific fields based on their respective literature bases, as well as to identify new trends, collaborative features, and research aspects (23, 24). While traditional review approaches must concentrate on a reasonable amount of literature, bibliometric techniques (together with meta-analyses) are particularly promising and appropriate to assist in understanding of the massive quantity of material (25). Furthermore, although bibliometric analysis depends more on quantitative approaches than conventional systematic literature reviews, it can prevent or at least lessen the impact of scholarly interpretation bias (24).

Although AI has changed various aspects of sports science, a bibliometric review regarding the applications of AI in tennis remains an under-explored topic. While several systematic reviews have explored various applications of ML in tennis (26–31), no bibliometric review has specifically examined the applications of AI in tennis. Therefore, the aim of this study was to conduct a comprehensive scoping and bibliometric review of articles on AI in tennis. The objectives were to analyze the chronological distribution of publications and citations, to map the network of contributing countries/regions and institutions, and to identify the most influential journals and authors in this research domain. This review aims to provide a clearer understanding of the trends, collaborations, and key contributors in the field of AI applications in tennis.

## 2 Methods

### 2.1 Data source and search strategy

With over 250 fields covered, the Web of Science (WoS) incorporates reliable worldwide citation databases spanning all areas of research (32). For several journals chosen according to the index, the WoS list offers comprehensive details on definitions, coverage remarks, and the most significant impact factor score (32). Numerous bibliometric studies, particularly those on sports, have made extensive use of it (33, 34). For this reason, the database used in this bibliometric review is the WoS Core Collection (WoS by Clarivate Analytics).

The search spanned until June 1, 2024, and several strategies were developed to account for the recent emergence of the topic. Utilizing a comprehensive approach, the search strategy entailed combining the term “tennis” with various AI-related terminologies using the Boolean operator “AND/OR” (35). These terminologies encompassed “artificial intelligence”, “machine learning”, “deep learning”, “neural network”, “support vector machine”, “nearest neighbor”, “random forest”, “Bayesian logistic regression”, and “predictive modeling”. This search strategy aimed to capture all relevant studies investigating the application of AI and its subfields, including machine learning, within the domain of tennis.

### 2.2 Inclusion and exclusion criteria

The following inclusion criteria were applied: (i) written in English; (ii) articles involving tennis; (iii) articles with data analyzed with ML algorithms, deep learning, or other AI techniques; (iv) articles related to, but not limited to, performance, health, match results, physiological data and other relevant aspects; (v) articles focused on tennis players, including humans and robots. Exclusion criteria were: (i) articles written in languages other than English; (ii) articles lacking information on critical information to ascertain the use of AI techniques; (iii) articles focused on other sports (i.e., football, basketball, etc.).

### 2.3 Screening process

Two reviewers examined the titles and abstracts of the selected papers independently. When an article's eligibility was questionable, the entire text was obtained. The same two reviewers evaluated the eligibility requirements and checked through the articles published in the integral. These reviewers conducted two independent rounds of evaluation for each article. The article's complete content was assessed after the title and abstract were assessed. Disputes involving eligibility were settled by conversation and, if required, with the help of a third reviewer.

**Figure 1:**
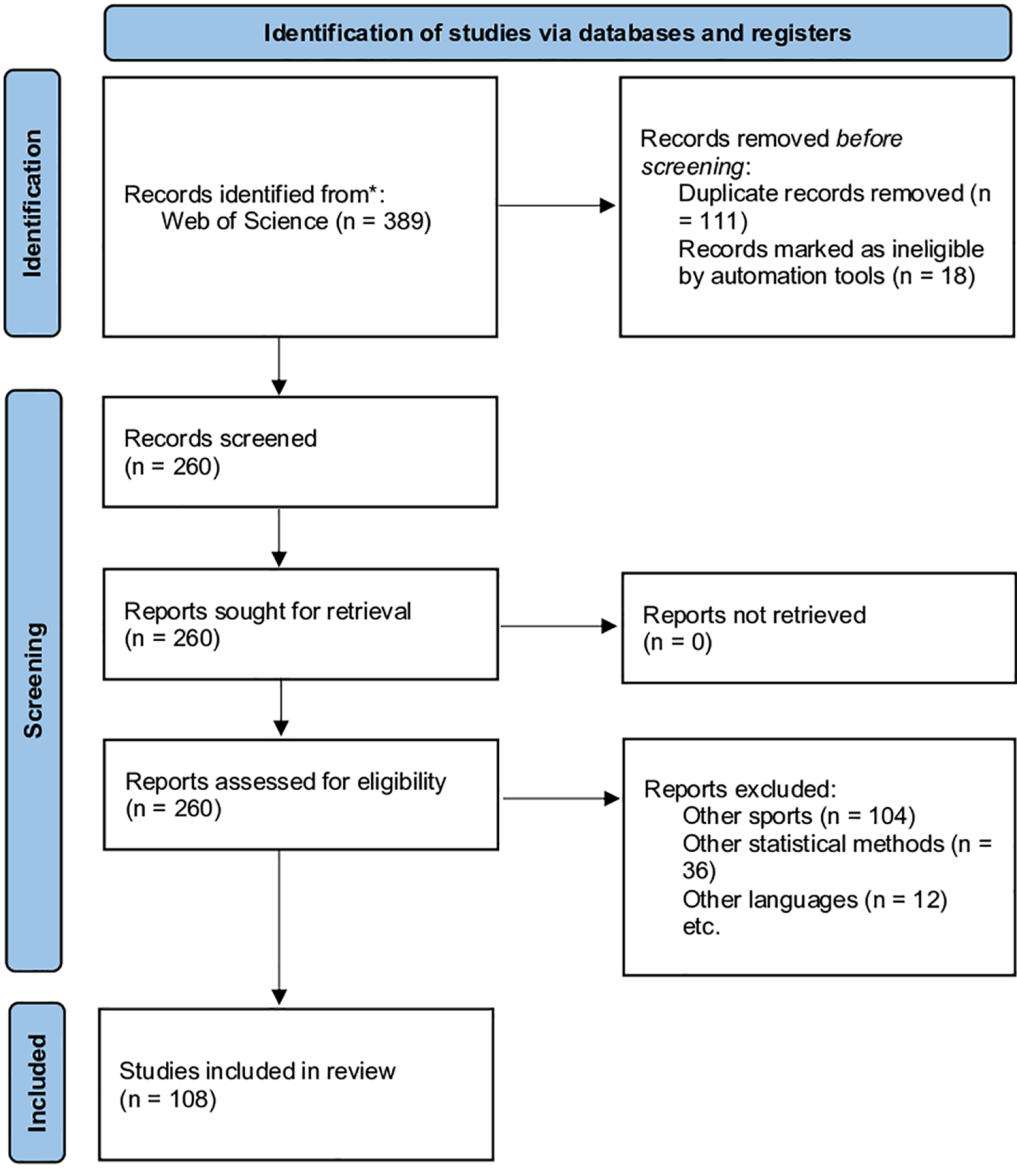
Flowchart of the review.

The WoS search yielded 389 records, of which 111 were duplicates and were therefore excluded. Additionally, 18 articles were excluded by automation tools (filtered to identify only articles written in English). The remaining 260 studies were then assessed by reading the relevant sections. 152 studies that did not meet the inclusion criteria (104 articles were focused on other sports, 36 articles used other statistical methods and 12 articles were in another language) were excluded. Finally, a total of 108 articles met the defined criteria and were included in the review. Figure 1 shows the identification, screening, and inclusion of the articles from the WoS database for the review. When full-text publications were accessible, further information was retrieved for an in-depth study, including the approach and results.

### 2.4 Analytical methods and tools

The bibliometric data was extracted and analyzed using specialized software. The measurement program VOSviewer (https://www.vosviewer.com), created by Van Eck and Waltman, is based on Java and is intended for use in the creation and display of bibliometric networks (36). These networks can be built based on citation, bibliographic coupling, co-citation, or co-authorship links, and they comprise journals, researchers, or individual publications. As a result, in the cooperative network visualization and the co-sponsorship network visualization, distinct clusters were visually represented by different colors, and collaborative relationships were indicated by lines linking nodes. Specifically, the average publication year graph's colors designate distinct years, making temporal analysis easier. The various densities are also reflected in the color spectrum, especially on density graphs where denser locations are shown by redder colors.

The assessment of individuals (primarily authors, institutions, journals, and countries) based on bibliographic information is included in the first category. Scientific mapping, which is the second category, examines the connections across disciplines, fields, specializations, individual articles, and authors through a geographical visual representation of bibliometric networks. Therefore, by examining the earlier categories in the VOSviewer program, a comprehensive evaluation of the applications of ML in tennis was completed.

The number of years after a document was published has a substantial correlation with its citation count (37). A manuscript that was published earlier will often have had more time to gain citations than one that was released recently. Consequently, raw citation counts are not a valid indicator of publication impact. For this reason, the citation analysis in this study was normalized to take into consideration variations in the years of publication, enabling a more precise evaluation of the influence of academic publications. To reduce the bias brought on by variations in publication dates in citation practices, normalization is crucial in bibliometric analysis (38). As a result, each article's total number of citations was divided by the publication's age, which was determined by subtracting the present year from the year of publication. Additional information regarding the calculation of the metric can be found in (39).

Additionally, using Excel's exponential smoothing, the number of papers expected to be published in the next ten years (until 2034) was estimated based on historical publication trends using “=FORECAST.ETS” function (Microsoft, Microsoft 365, Washington, USA) (40). This makes it possible to evaluate time-series data and generate forecasts or predictions based on previous trends.

## 3 Results

### 3.1 Progression of publications by year

**Figure 2:**
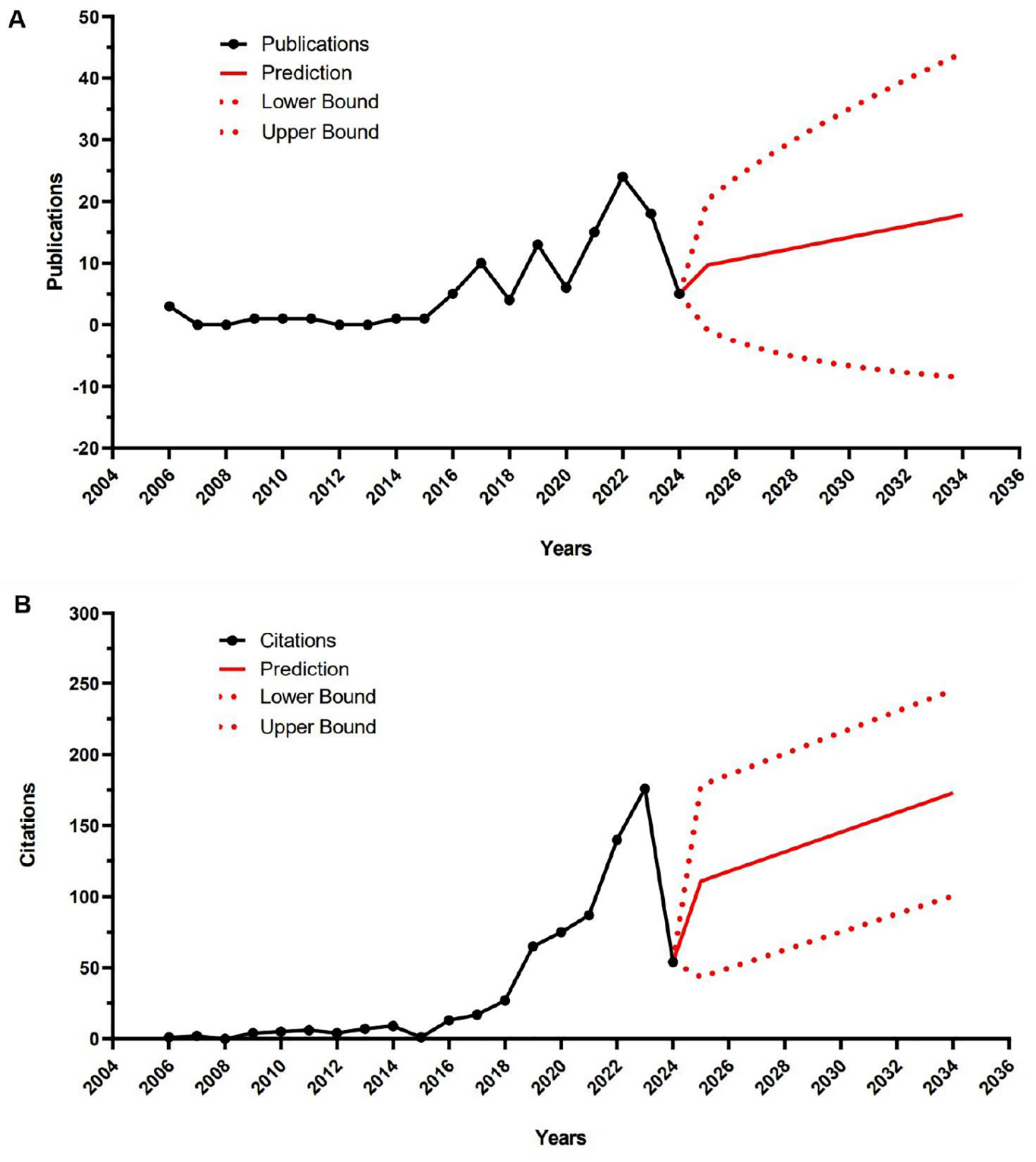
Publication output on AI in tennis by year and estimated number of articles for the next 10 years. The red solid line represents the estimate, and the red dashed lines represent the 95% confidence intervals **(A)**; Citation outputs related to AI in tennis research by year and the estimated number of citations for the next 10 years. The red solid line represents the estimate and the red dashed lines the 95% confidence intervals **(B).**

Between 2006 (the year of the first publication with ML in tennis) and 2024, the WoS database contained a total of 108 articles related to AI in tennis. Figure 2A shows the publication output regarding AI in tennis research during the period from 2006 to 2024. Figure 2B shows the citation output regarding AI in tennis research during the period from 2006 to 2024. The yearly publication trends reveal intermittent gaps in certain time intervals with no publications, particularly in the years 2007–2008 and 2012–2013. Despite these gaps, there is an overall upward trend in publications on the topic until 2022. After 2022, however, there is a noticeable decline in annual publications, with 24 records in 2022, 18 in 2023, and 5 in 2024. Notably, the largest number of articles was published in 2022, with 24 articles focusing on AI in tennis.

Regarding the yearly citation trends demonstrate intermittent gaps in certain time intervals with no citations, particularly in 2008. Despite these gaps, there is a clear upward trend in citations on the topic until 2023. After 2023, however, there is a marked decrease in annual citations, with 176 citations in 2023 and 54 in 2024. Notably, the largest number of citations was recorded in 2022, with 140 citations related to ML in tennis.

The exponential smoothing estimation model showed an average of 13.36 ± 2.74 articles per year (95% confidence intervals: −5.30; 32.01) that may be published between 2025 and 2034. For citations, the estimation model indicated an average of 139.14 ± 20.88 citations (95% confidence intervals: 69.47; 208.82) per year between the same time periods.

### 3.2 Web of science (WoS) categorization

**Figure 3:**
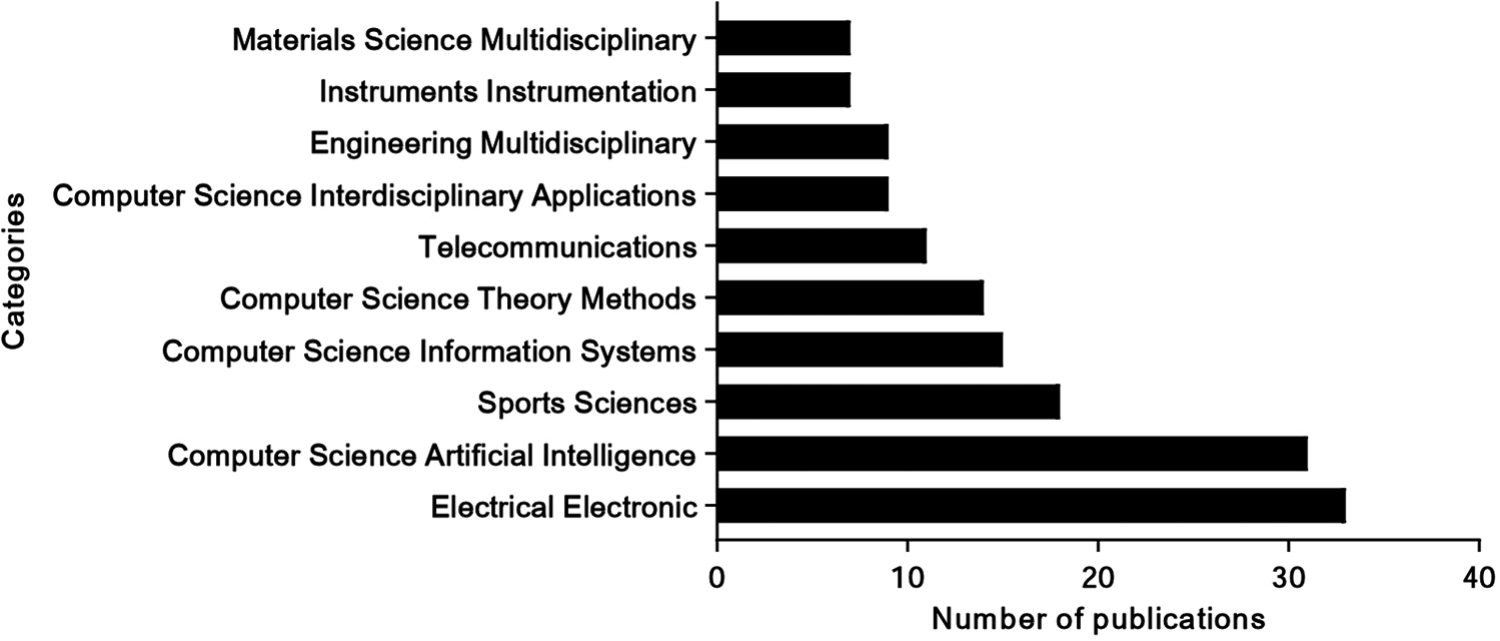
Top 10 Web of science categories for AI in tennis research.

The study field can be categorized, and potential interdisciplinary linkages might be found by examining the categories within the WoS. Figure 3 presents the analysis of the WoS categories. The top-ranking fields are Engineering Electrical Electronic (*n* = 33 publications), Computer Science Artificial Intelligence (*n* = 31 publications), Sports Sciences (*n* = 18 publications), Computer Science Information Systems (*n* = 15 publications), Computer Science Theory Methods (*n* = 14 publications), Telecommunications (*n* = 11 publications), Computer Science Interdisciplinary Applications (*n* = 9 publications), Engineering Multidisciplinary (*n* = 9 publications), Instruments Instrumentation (*n* = 7 publications), Materials Science Multidisciplinary (*n* = 7 publications).

### 3.3 Analysis of countries/regions and institutions

A total of 34 countries and regions have contributed to ML in tennis research, according to the country of the corresponding author. In particular, the historical perspective highlights the development of global research. In the first five years after the publication of the first article, ML in tennis was led by only six countries (People's Republic of China, Singapore, France, Slovenia, Thailand and Tunisia).

Over the next five years, however, researchers from eight countries/regions (England and India), along with the aforementioned People's Republic of China, Singapore, France, Slovenia, Thailand and Tunisia, significantly expanded their involvement.

**Figure 4:**
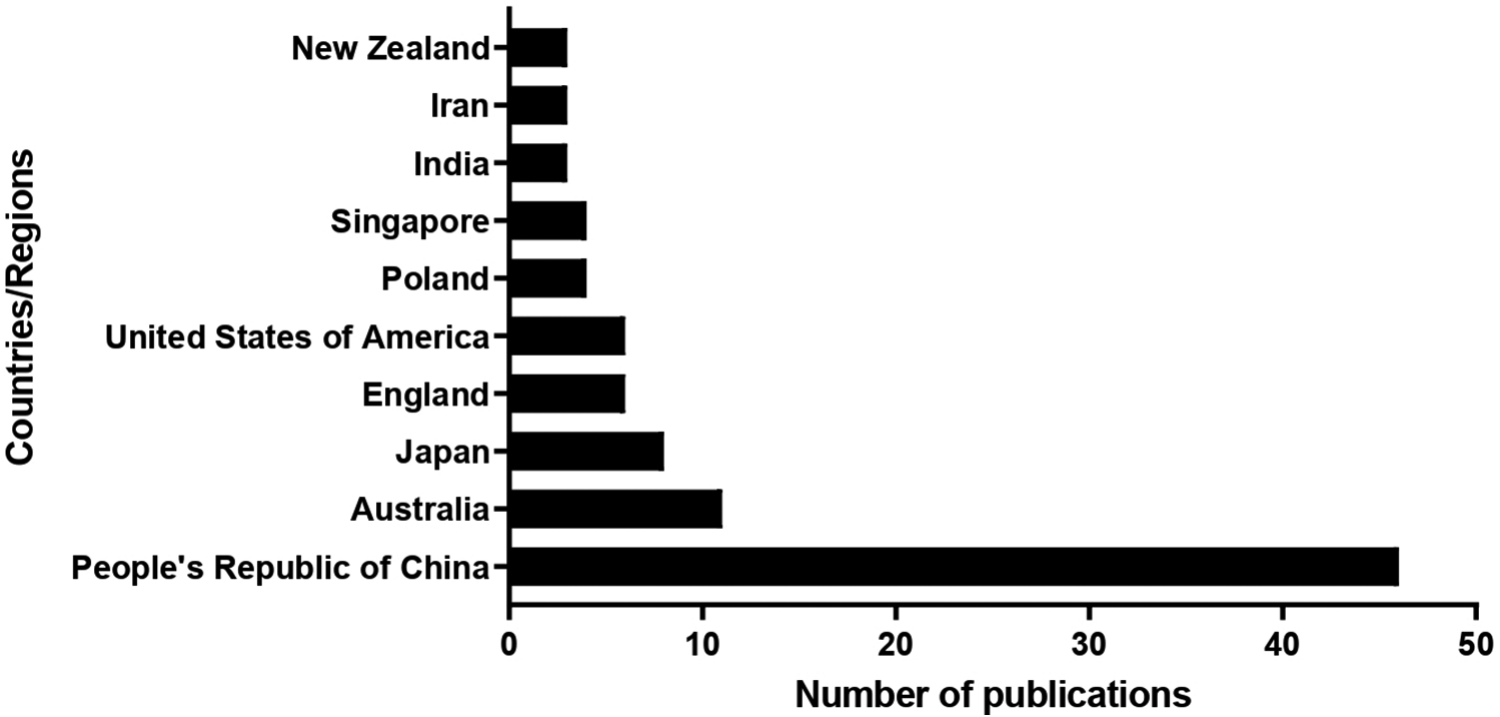
The top ten countries/regions in AI and tennis research.

The distribution of the number of articles by country/region is shown in Figure 4. The top ten countries and regions were People's Republic of China (*n* = 46 publications) with 39% of the total publications, followed by Australia (*n* = 11 publications), Japan (*n* = 8 publications), England (*n* = 6 publications), USA (*n* = 6 publications), Poland (*n* = 4 publications), Singapore (*n* = 4 publications), India (*n* = 3 publications), Iran (*n* = 3 publications), and New Zealand (*n* = 3 publications).

Co-authorship cluster analysis, which determines the relatedness of articles based on the number of co-authored documents, was performed on 11 countries/regions that produced at least 3 articles from the 34 countries/regions that published articles about AI in tennis and had international collaboration among their authors.

**Figure 5:**
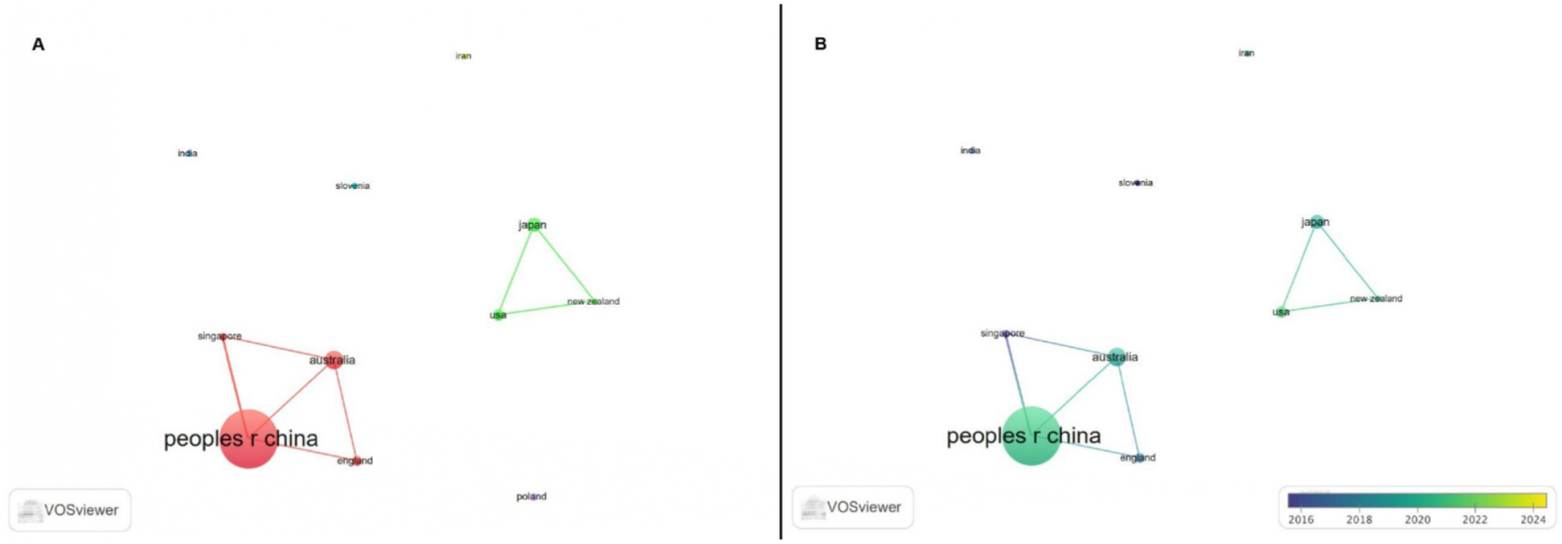
The cooperation network visualization map of institutions based on VOSviewer **(A)**; The cooperation network visualization map of institutions based on VOSviewer with the timeline **(B).**

Figure 5 shows the visualization map of the collaboration network created by VOSviewer (panel A) and the visualization map of the timeline network created by VOSviewer (panel B).

According to the results of the clustering analysis, six different clusters were formed: cluster 1: Australia, England, Peoples r China, and Singapore; cluster 2: Japan, New Zealand and USA; cluster 3: India; cluster 4: Iran; cluster 5: Poland and cluster 6: Slovenia. In addition, the total link strength scores were calculated, indicating the strength of cooperation among the countries. The top 10 countries/regions with the highest total link strength scores were: Peoples r China = 5, Singapore = 4, Australia = 3, England = 2, Japan = 2, New Zealand = 2 and USA = 2. The remaining countries/regions have a total link strength of 0, indicating that they do not form a cluster with other countries.

### 3.4 Analysis of journals and conferences chapters

A total of 121 journals and conferences were involved in publishing about AI in tennis. The top ten journals and conferences chapters are: Applied Sciences Basel (*n* = 5 publications), Sensors (*n* = 4 publications), Computational Intelligent and Neuroscience (*n* = 3 publications), IEEE Access (*n* = 3 publications), Journal of Sports Sciences (*n* = 3 publications), Lecture Notes in Computer Science (*n* = 3 publications), Soft Computing (*n* = 3 publications), Wireless Communications Mobile Computing (*n* = 3 publications), Advances in Nano Research (*n* = 2 publications), and IEEE Computer Society Conference On Computer Vision And Pattern Recognition Workshops (*n* = 2 publications).

### 3.5 Analysis of authors

Since 2006, 310 researchers have contributed to the advancement of research in this specific topic. The use of visualization maps can provide valuable insights into potential collaborators, helping researchers to establish productive partnerships.

**Figure 6:**
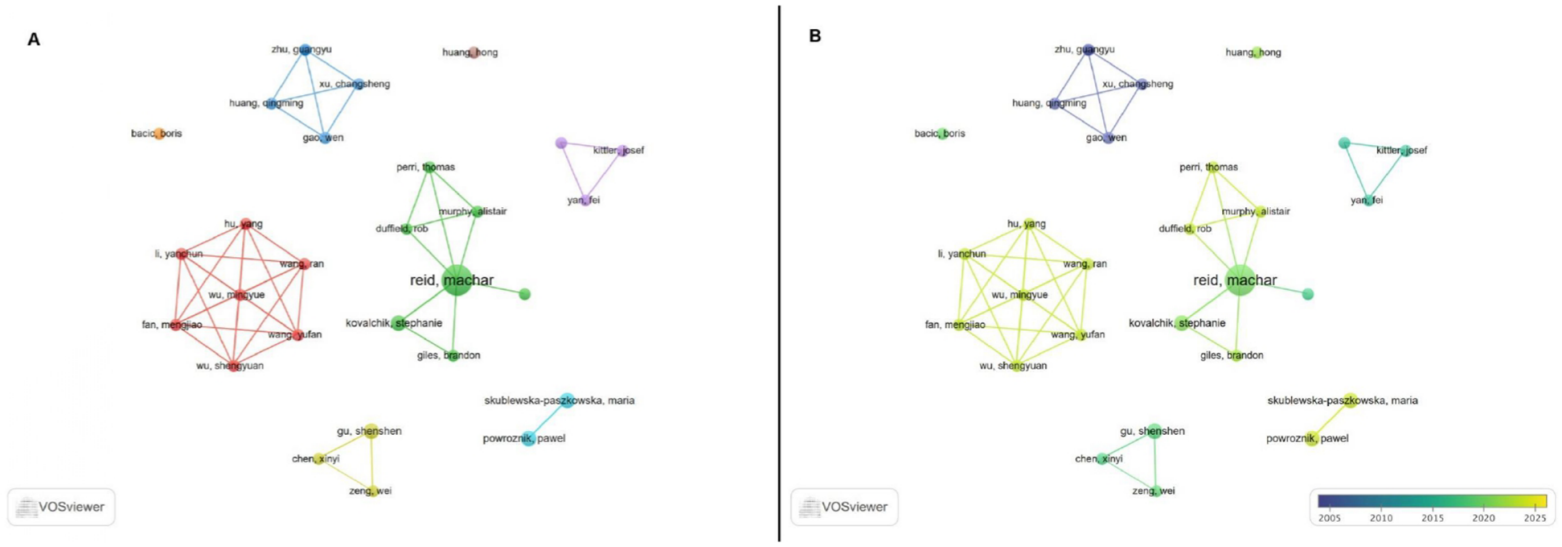
The cooperation network visualization map of authors based on VOSviewer **(A)**; The cooperation network visualization map of authors based on VOSviewer with timeline **(B).**

Using a threshold of 2 documents per author, Figure 6 allows the visualization of eigth distinct clusters. As shown in the figure, the research landscape in this domain is mainly a divided network of small clusters. The presence of two clusters constituted by seven members participating in the collaboration is noteworthy.

The top 10 most active authors, based on the number of documents, are Reid M. (*n* = 8), Gu SS. (*n* = 3), Kovalchik S. (*n* = 3), Powroznik P. (*n* = 3), Skublewska-paszkowska M. (*n* = 3), Bacic B. (*n* = 2), Chen XY. (*n* = 2), Duffield R. (*n* = 2), Fan MJ. (*n* = 2) and Gao W. (*n* = 2).

### 3.6 Analysis of keywords

In combination keyword clustering can be used to identify hot spots in the field of study as well as new trends and patterns in a topic's development. It can provide insight into an academic field's internal structure as well as its research frontier.

**Figure 7:**
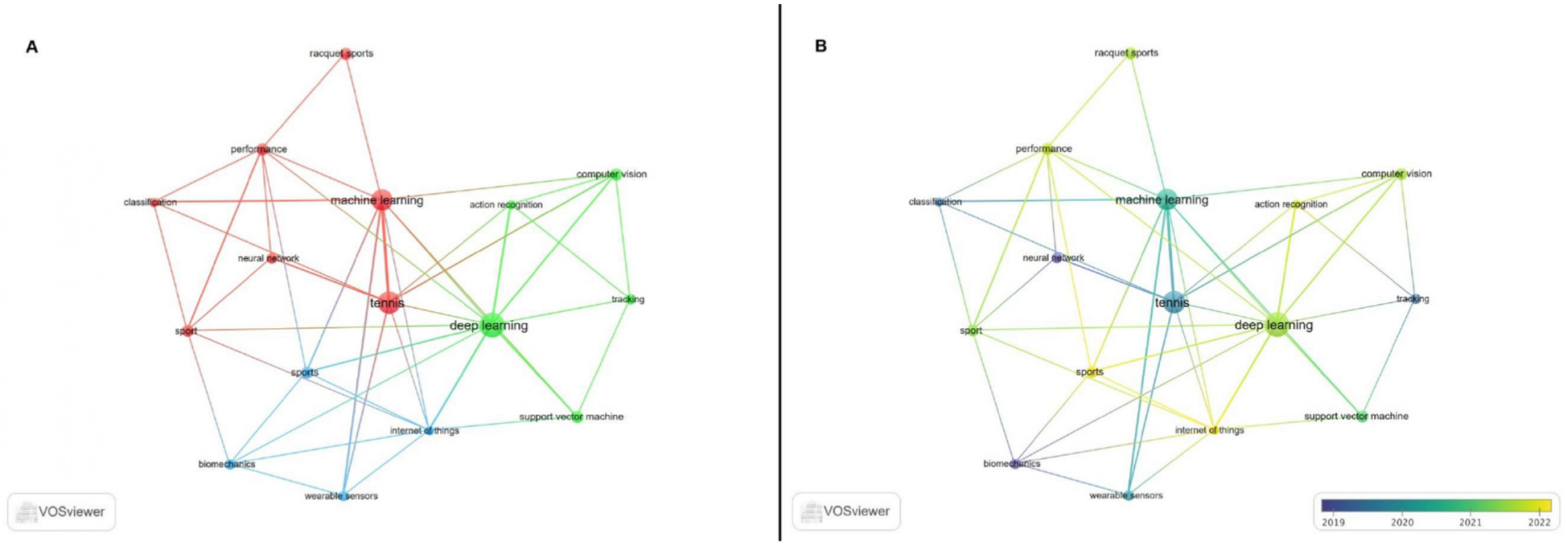
Keywords clustering map based on VOSviewer **(A)**; Keywords clustering map with the timeline based on VOSviewer **(B).**

The co-occurrence keyword analysis is displayed in Figure 7. Different clusters can be seen if a threshold of four occurrences per keyword is applied. More occurrences and more representativeness of the hotspots in the field are indicated by larger dots. The more lines that indicate the number of times two keywords occur in the same article, the stronger the relationship is between the nodes. The various colors stand for various clusters, or study topics, and the blue to yellow color scale indicates the appearance time. Therefore, the clusters emerged with the following keywords: cluster 1 (classification, machine learning, neural network, performance, racquet sports, sport and tennis); cluster 2 (action recognition, computer vision, deep learning, support vector machine and tracking); cluster 3 (biomechanics, internet of things, sports and wearable sensors). Table 1 shows the link strength of each keyword and the number of occurrences.

**Table 1:** Keyword's link strength and number of occurrences in AI and tennis research.

Keyword	Occurrences	Total Link Strength
Classification	4	5
Machine learning	14	20
Neural network	5	4
Performance	6	8
Racquet sports	6	2
Sport	6	7
Tennis	15	17
Action recognition	4	6
Computer vision	6	7
Deep learning	17	18
Support vector machine	6	5
Tracking	5	4
Biomechanics	5	5
Internet of things	4	10
Sports	6	7
Wearable sensors	5	7

### 3.7 Analysis of references

Since 2006, publications in this area have been cited 627 times in total without self-citations. The top five normalized cited references are detailed in Table 2, which shows the average citations per year for each article. Notably, the article with the highest normalized citation value is by the author Whiteside et al. with the article entitled “Monitoring Hitting Load in Tennis Using Inertial Sensors and Machine Learning” (41).

**Table 2:** Top five cited references in AI and tennis research detailing the citation index.

Rank	Title	First author (year)	Citation Index
1.	Monitoring Hitting Load in Tennis Using Inertial Sensors and Machine Learning (41)	Whiteside et al. (2017)	8.43
2.	Deep historical long short-term memory network for action recognition (42)	Cai et al. (2020)	7.25
3.	Differentiating movement styles in professional tennis: A machine learning and hierarchical clustering approach (43)	Giles et al. (2023)	7
4.	A machine learning approach for automatic detection and classification of changes of direction from player tracking data in professional tennis (44)	Giles et al. (2020)	6.75
5.	RNN-Based Quadratic Programming Scheme for Tennis-Training Robots with Flexible Capabilities (45)	Jin et al. (2023)	6

## 4 Discussion

### 4.1 Comprehensive analysis

The aim of this study was to conduct a comprehensive scoping and bibliometric review of articles using AI in tennis. From the bibliometric analysis of the use of AI in tennis, it is evident that there has been an unstable yet overall upward trend in research publications and citations. The analysis identified intermittent gaps in publication output during certain intervals, notably in the years 2007–2008 and 2012–2013, which reflects variability in research activity. Despite these gaps, the peak in publication numbers occurred in 2022 with 24 articles, while citation activity reached in 2023 176 citations. This suggests increased research interest and recognition of the importance of AI in tennis during these years. The subsequent decline in both publications and citations post 2022 indicates a potential change. However, the exponential smoothing estimation model predicts an average of 13.36 ± 2.74 articles per year and 139.14 ± 20.88 citations per year between 2025 and 2034, suggesting sustained interest in AI research in tennis in the coming years.

Additionally, the Web of Science categorization reveals that AI in tennis research spans multiple disciplines, with the most prominent fields being Engineering Electrical Electronic, Computer Science Artificial Intelligence, and Sports Sciences. This interdisciplinary approach underscores the comprehensive impact of AI technologies in various aspects of tennis, from performance analysis to equipment innovation.

Furthermore, a total of 34 countries have contributed to research, showcasing a global collaborative effort. Notably, China has emerged as a leading contributor, with significant input also from countries such as Australia, Japan, and the United States. This geographic distribution highlights the global recognition of the potential benefits of AI in enhancing tennis performance and strategy.

### 4.2 Keywords analysis and research hotspots

This bibliometric review offers critical insights into the evolving landscape of AI research in tennis, focusing on identifying emerging research hot spots through a comprehensive keyword analysis. The analysis identifies three primary clusters, each representing a significant concentration of research efforts and advancements in AI applications within tennis. These clusters not only map the current research landscape but also indicate promising directions for future studies, marking them as research hot spots.

#### 4.2.1 Performance analysis and optimization (cluster 1)

The first cluster is characterized by keywords such as classification, ML, neural network, performance, racquet sports, sport, and tennis. This cluster underscores the pivotal role of AI in performance analysis and optimization. Research in this area leverages sophisticated ML algorithms and neural networks to classify player movements, predict performance outcomes, and refine training regimens (46–48). The specific focus on tennis highlights the tailored application of these technologies to enhance athlete performance. Studies within this cluster emphasize the development of data-driven insights and personalized training strategies, essential for maintaining a competitive advantage in the sport (49, 50). The advancements in this area are crucial for developing more precise and effective performance enhancement techniques, enabling athletes and coaches to optimize training programs based on evidence and predictive analytics.

Recent developments in AI, particularly in ML and neural networks, have allowed for more detailed and accurate performance analysis (51–53). The ability to classify and analyze specific movements and techniques offers invaluable feedback for athletes, enabling them to refine their skills and strategies. Additionally, the predictive capabilities of AI can help anticipate performance trends and outcomes, allowing for proactive adjustments in training and competition strategies.

#### 4.2.2 Technological integration and innovation (cluster 2)

The second cluster is defined by keywords such as action recognition, computer vision, deep learning, support vector machine, and tracking. This cluster focuses on the integration of advanced AI technologies into tennis. Research aims to develop sophisticated systems for action recognition and tracking, employing techniques like support vector machines to improve accuracy and efficiency (54–57). These innovations enable real-time analysis of player movements and game dynamics, providing critical feedback for performance enhancement. The application of cutting-edge technologies such as computer vision and deep learning reflects the sport's adaptation to modern advancements, bridging traditional methods with progressive AI solutions.

In particular, computer vision technology has revolutionized the way player movements and game dynamics are analyzed. By processing video footage in real-time, these systems can track player positions, movements, and interactions with unprecedented precision. This capability not only enhances performance analysis but also facilitates the development of strategic insights that can be applied during matches. Deep learning algorithms further enhance these systems by improving the accuracy and reliability of action recognition and tracking (58–60). The integration of these technologies into training and competition settings represents a significant advancement in the sport, providing players and coaches with powerful tools to enhance performance and strategy.

#### 4.2.3 Biomechanics and wearable technology (cluster 3)

The third cluster is characterized by keywords such as biomechanics, sports, and wearable sensors. This cluster highlights the connection between biomechanics and wearable technology. Research in this area focuses on the use of wearable sensors to collect detailed biomechanical data, which is then analyzed to monitor player performance (61, 62). This cluster is particularly relevant for injury prevention and rehabilitation, as well as for optimizing training techniques based on biomechanical insights. The advancements in this field signify a move towards more comprehensive and real-time monitoring of athletes, enhancing both performance and safety.

Moreover, wearable technology has become increasingly sophisticated, with sensors capable of capturing a wide range of biomechanical data, including movement patterns, muscle activity, and joint angles (63). This data provides a detailed understanding of the physical demands and stresses placed on athletes during training and competition. By analyzing this information, researchers and trainers can identify potential injury risks and develop targeted interventions to prevent injuries and enhance recovery. This holistic approach to performance monitoring and injury prevention is essential for maintaining athlete health and longevity in the sport.

## 5 Implications for the future

The identification of these clusters through keyword analysis underscores the dynamic and evolving nature of AI applications in tennis. As the sport continues to bridge tradition with advancement, the insights from these clusters offer several key implications for the future.

First, the ongoing research in performance analysis and optimization will continue to provide athletes and coaches with valuable data-driven insights, fostering personalized and effective training methodologies. The advancements in ML and neural networks will enhance the ability to classify and predict performance, enabling more precise and effective training interventions. The integration of computer vision and deep learning technologies will pave the way for innovative solutions that enhance real-time performance monitoring and feedback, transforming traditional training and game analysis. These technologies will provide players and coaches with actionable insights that can be applied during matches, improving strategic decision-making and performance outcomes.

A continued exploration of these identified hot spots will further solidify the role of AI in tennis, supporting interdisciplinary collaborations and the development of novel applications in sport. The integration of AI technologies into tennis represents a significant advancement in the sport, offering new opportunities for research and innovation that align with the sport's history.

## 6 Conclusions

This bibliometric review allowed us to understand the evolution of articles published on the use of AI in tennis. Although the research activity has shown intermittent gaps, there has been a consistent increase in publications and citations, particularly peaking in 2022 and 2023. The prediction model indicates that the number of articles and citations on this topic will continue to grow until 2034. Three main research clusters were identified (Performance Analysis and Optimization, Technological Integration and Innovation, and Biomechanics and Wearable Technology). These clusters highlight AI's pivotal role in enhancing performance, integrating advanced technologies, and improving biomechanics. The ongoing research in these areas will continue to drive innovation and interdisciplinary collaboration in tennis.
